# Meet us on the phone: mobile phone programs for adolescent sexual and reproductive health in low-to-middle income countries

**DOI:** 10.1186/s12978-016-0276-z

**Published:** 2017-01-17

**Authors:** Nicole B. Ippoliti, Kelly L’Engle

**Affiliations:** 1 Global Health, Population, and Nutrition, FHI 360, 359 Blackwell Street, Suite 200, Durham, NC 27701 USA; 2 School of Nursing and Health Professions, University of San Francisco, California, 2130 Fulton Street, San Francisco, CA 94117 USA

**Keywords:** Adolescents, Sexual and reproductive health, mhealth, Mobile phones, LMIC

## Abstract

**Introduction:**

mHealth as a technical area has seen increasing interest and promise from both developed and developing countries. While published research from higher income countries on mHealth solutions for adolescent sexual and reproductive health (SRH) is growing, there is much less documentation of SRH mHealth interventions for youth living in resource-poor settings. We conducted a global landscape analysis to answer the following research question: How are programs using mHealth interventions to improve adolescent SRH in low to middle income countries (LMICs)?

**Methods:**

To obtain the latest information about mHealth programs targeting youth SRH, a global call for project resources was issued in 2014. Information about approximately 25 projects from LMICs was submitted. These projects were reviewed to confirm that mobile phones were utilized as a key communication media for the program, that youth ages 10–24 were a prime target audience, and that the program used mobile phone features beyond one-on-one phone calls between youth and health professionals.

**Results:**

A total of 17 projects met our inclusion criteria. Most of these projects were based in Africa (67%), followed by Eurasia (26%) and Latin America (13%). The majority of projects used mHealth as a health promotion tool (82%) to facilitate knowledge sharing and behavior change to improve youth SRH. Other projects (18%) used mHealth as a way to link users to essential SRH services, including family planning counseling and services, medical abortion and post-abortion care, and HIV care and treatment. There was little variation in delivery methods for SRH content, as two-thirds of the projects (70%) relied on text messaging to transmit SRH information to youth. Several projects have been adapted and scaled to other countries.

**Discussion:**

Findings suggest that mHealth interventions are becoming a more common method to connect youth to SRH information and services in LMICs, and evidence is emerging that mobile phones are an effective way to reach young people and to achieve knowledge and behavior change. More understanding is needed about the challenges of data privacy and phone access, especially among younger adolescents, and the role that mHealth solutions for adolescent SRH should play in health programming for young people.

**Electronic supplementary material:**

The online version of this article (doi:10.1186/s12978-016-0276-z) contains supplementary material, which is available to authorized users.

## Plain English summary

The use of mobile phones to transmit health information and provide links to services to improve health behaviors among hard to reach populations has gained increasing attention in recent years. A global review was conducted to assess the extent to which programs leveraged mobile phones to improve youth sexual and reproductive health (SRH) in low to middle income countries. To obtain the latest information about programs that used mobile phones to improve youth SRH, a global call for project resources was issued in 2014. Approximately 25 projects were submitted for review, of which 17 projects were selected for inclusion in this review. Findings suggest that within these projects, mobile phones were commonly used to encourage youth to seek health services and to transmit SRH information to youth to increase their knowledge and promote positive health behavior. Most projects used text messaging to transmit information to youth. While findings suggest that these interventions are an efficient and appealing way to connect users to needed SRH information and services, more information is needed about how to ensure the privacy of young people who might be sharing their phones with others.

## Background

### Youth and technology

Young people represent the highest proportion of global consumers of mobile technology. Globally, more than 93% of the world’s population is covered by mobile phone networks, and more than 87% of people living in the developing world are mobile phone subscribers [1]. Due to declining mobile phone costs and increasing reliance on mobile phones as essential commodities, mobile phone use is on the rise, even in the most resource-poor settings. Data on mobile phone penetration among younger populations is limited, but recent findings suggest that mobile phone ownership among youth in low-to-middle income countries (LMICs) is steadily increasing [2]. A 2014 survey of 24 emerging nations revealed that between 53 and 95% of people aged 18–29 owned mobile phones; the overall average was nearly 83% ownership among young people [3]. Text messaging is found to be the most popular form of mobile phone communication, particularly among young people [3]. A recent study of seven African countries (South Africa, Nigeria, Senegal, Kenya, Ghana, Tanzania, and Uganda) documented that texting was substantially more popular among individuals ages 18–34 than those 35 and older [4].

### Mobile phones and reproductive health

mHealth—the use of mobile phones to improve health behaviors and services—is a promising approach in global health that is increasingly applied to link young people to health information and services. Privacy, convenience, and access—hallmarks of mobile phones—make them especially appealing to adolescents. Global reproductive health programs are leveraging mobile phones (including all mobile phones, and not just limited to smart phones) to support adolescents’ health in rural and urban communities, through diverse communication formats that link young people to information and services on a wide-range of sexual and reproductive health (SRH) topics. In LMICs, mobile phones provide cost-effective, efficient, and a highly suitable communication channel for reaching and engaging youth around SRH issues. This brief provides programmatic examples of mHealth interventions designed to improve adolescent SRH in LMICs in order to inform health and technology practitioners about the ways in which mobile solutions can be applied to advance adolescent SRH outcomes in low-resource settings. By highlighting these projects, we aim to fill an important information gap on the modalities, content, successes, and barriers faced during mHealth project implementation for adolescent SRH in LMICs.

mHealth overcomes many barriers experienced by young people when seeking SRH information and services. Youth commonly report stigma and discrimination from health care workers when seeking SRH services, along with cost prohibitions and transportation challenges in reaching heath facilities [5–8]. Embarrassment and perceived lack of privacy and confidentiality in communications with adults around SRH add to youths’ challenges in obtaining high-quality, comprehensive information [5, 6]. Yet, adolescents lack information and have misconceptions about reproductive health, contraception and condoms, and HIV [7–9]. In this challenging landscape of information provision, programs that provide alternative, but complementary, means of reaching young people are sorely needed. mHealth programs offer information and meaningful engagement to young people outside of facility settings to discretely deliver relevant and timely content without stigma or judgment.

Offering SRH information via mobile technologies has an emerging evidence base to recommend mHealth as an acceptable, feasible, and promising intervention approach in resource-poor settings [10–13]. Global research suggests that providing SRH information via mobile phones is highly appealing to young people and can positively influence their SRH outcomes including improving knowledge, reducing sexual risk behavior, and increasing utilization of health services [2, 14–17]. Finally, data from costing studies suggest that mHealth program expenses are reasonable and affordable, and text messaging in particular may be more cost-effective than other communication strategies [18–20]. The appealing qualities of mHealth interventions have translated into growing recognition that mobile phones offer a promising platform for reaching large numbers of adolescents across diverse settings with private, essential, high-quality, and comprehensive SRH information and support.

## Methods

To obtain the latest information about mHealth programs targeting adolescent SRH, a call for project resources (Additional file 1) was issued in July 2014. The call for resources was widely promoted via social media, relevant listservs, and partner websites. Information about approximately 25 different projects was submitted, and program staff from each project were contacted to clarify the information and confirm program details. Projects were reviewed to ensure that the program aim was to improve SRH, and that the program primarily targeted or was accessed by youth ages 10-24. Projects were also reviewed to confirm that mobile phones were utilized as a key communication media for the program, and that the program used mobile phone features beyond one-on-one phone calls between youth and health professionals.

After review, a final total of 17 projects was selected on the basis of meeting the eligibility criteria described above, and because they targeted young people in low and middle income countries. For each project, data were extracted from project slide presentations, case studies and technical briefs, project websites, project reports, and personal communications with project managers to describe the following features of the mobile phone project: project name and sponsoring organization, geographic location, project design, type of mhealth application used, target population, duration of the project, and SRH knowledge, attitude, and behavior outcomes targeted by the mHealth project. Data on the projects’ strategies for reaching young people as well as the aim of the projects also were extracted to detail the methods, approaches, and populations that these mHealth projects in LMICs address.

## Results

The 17 projects that met the inclusion criteria are summarized in Table 1. These projects revealed diverse geographic representation, content, and delivery methods among programs that used mobile technology to improve the SRH of young people in LMICs. The majority of these interventions were based in Africa (67%), followed by Eurasia (26%) and Latin America (13%).

**Table 1:** Descriptions of mHealth projects for young people

Project Name and Organization	Country	Purpose	Strategies for Reaching Young People	Project Aim
InfoAdo Senegal, One World	Senegal	Mobile phone question and answer service by trained counsellors from local organizations provide rapid, accurate, and non-judgmental answers. Addresses adolescent SRH issues for in and out of school youth ages 11–34.	SMS and web-based question-and-answer service	Health Promotion
Chat Salud Nicaragua, Chat Salud	Nicaragua	Mobile phone question and answer service that delivers gender-based violence, contraception, sexual health, and STI information.	Opt-in, menu-based, two-way SMS platform.	Health Promotion
CHITCHAT Belize, CHITCHAT	Belize	Hotline service that provides SRH information for ages 15–45 that is accurate, culturally relevant and aimed at dispelling popular misconceptions/myths about reproductive health and family planning.	SMS-based info hotline	Health Promotion
E-voucher, Marie Stopes	Ethiopia	A subsidized voucher program to increase young people ages 15–29’s access to and choice of contraceptive methods. Vouchers can be redeemed at one of the health facilities attached to the program to receive a free counselling session on all contraceptive methods to ensure voluntary and informed choice.	Randomly assigned voucher codes are sent directly to the client’s mobile phone.	Links to Services
Weleli Info Ado, One World	Mali	Through eLearning environment, a mobile phone question and answer service, InfoAdo Mali addresses adolescent SRH issues for young people ages 11–34 in and out of school. Trained counsellors from local organizations provide rapid, accurate, and non-judgmental answers.	SMS and web-based question-and-answer service	Health Promotion
Learning about Living Cambodia, One World	Cambodia	Addresses adolescent SRH issues for young people ages 11–34 in and out of schools. Trained counsellors from local organizations provide rapid, accurate, and non-judgmental answers.	SMS and web-based question-and-answer service and mobile podcasts.	Health Promotion
Bila7araje Morocco, One World	Morocco	Addresses adolescent SRH issues for young people ages 11–34 in and out of schools. Trained counsellors from local organizations provide rapid, accurate, and non-judgmental answers.	Mobile phone (SMS and emails), a web-based question-and-answer service, and Facebook chat.	Health Promotion
Ma3looma, One World	Egypt	Addresses adolescent SRH issues for young people ages 10–29 in and out of schools. Trained counsellors from local organizations provide rapid, accurate, and non-judgmental answers.	Mobile phone (SMS and emails), a web-based question-and-answer service, and Facebook chat.	Health Promotion
m4RH, FHI 360	Tanzania, Kenya, Uganda	SRH and family planning information service. 82% of users are ages 29 or younger.	Opt-in, menu-based, two-way SMS program which also include role model stories of FP use.	Health Promotion & Links to Services
m4Youth, Pathfinder	Ethiopia	Tailored SRH, family planning, and HIV information tor university students and peer educators.	A menu-based SMS service, which students enroll by texting a short code to receive messages containing SRH menu options.	Health Promotion
m-ASSIST, Cell Life	South Africa	Mobile phone solution to improve provision of medical abortion and post abortion family planning use to youth 18–29 in South Africa with 50% of users ages 18–24.	SMS messages were sent to (1) coach women through the abortion process (2) how to manage side effects (3) encourage post abortion family planning use	Links to Services
mCENAS, Pathfinder	Mozambique	A SMS-based role model story showing common barriers to contraceptive use faced by youth, as well as an informational message system aimed at increasing knowledge about contraceptive methods for youth 15–24.	Role model stories and answers to FAQs via text messages on family planning access and uptake	Health Promotion
mCHAT, Save the Children	Thailand	Use of social media websites, online forums, and mobile apps to disseminate information on HIV prevention, treatment, care and support to young men who have sex with men (MSM) and transgender people.	Social media/ mobile apps	Health Promotion
menZDRAV Foundation	Russia	Deliver confidential and accurate SRH information and HIV counseling to MSM aged 18–25 years living with HIV.	Facebook app and Skype	Health Promotion & Links to Services
My Question, Population Council and Learning about Living	Nigeria	SRH information and education delivery service for young adults. Trained counselors provide responses using a database of answers to frequently asked questions or customized replies.	SMS and web-based question-and-answer service	Health Promotion
Project Khuluma, SHM Foundation	South Africa	Provide psychosocial support to HIV positive adolescents ages 13–18.	Provides tailored psychosocial support via voice and SMS to adolescents with HIV	Adherence
Yangpela Hotline, Marie Stopes	Papa New Guinea	Confidential, youth-friendly SRH information for young people ages 15–24	SMS and voice-based info hotline	Health Promotion

### m-Health delivery methods

Most projects (70% or 12 projects) relied on text messaging to transmit SRH information to their users (Table 1). These programs demonstrate the wide utility of SMS as a way to transmit and facilitate knowledge sharing within various domains of adolescent SRH. Programs such as mCENAS, M-ASSIST, and Project Khuluma highlight ways in which SMS was leveraged to transmit information, support, and counseling on different SRH services. Both M-ASSIST and Project Khuluma used SMS to transmit messages on post-abortion care and psychosocial support for youth living with HIV, respectively. m4RH and mCENAS also used SMS functionalities to transmit role model stories about contraceptive use and decision-making. These examples suggest that the functionality of SMS is responsive to innovative ways of health messaging, including direct text messages, theory-based role model story narratives, psychosocial support, and counselling.

The remaining interventions (5 projects) employed a mix of informational hotlines or social media applications to reach their users. Notably, Marie Stopes in Ethiopia sent e-vouchers directly to the user’s mobile phone, redeemable at affiliated health facilities for free counselling sessions on contraceptive methods, as well as medical abortion and post-abortion care services. menZDRAV offers anonymous HIV counseling and support via social media and Skype to men who may be reluctant to attend support groups for fear of having their sexual orientation or HIV status publicly identified.

Age segmentation data of users extracted from the interventions suggests that older adolescents (ages 15 and above) tend to be the primary beneficiaries of these mHealth interventions, with lower program uptake among younger adolescents.

### Intervention strategies for reaching young people

Most interventions (82% or 14 projects) used mHealth as a health promotion tool to facilitate knowledge sharing and behavior change. These programs provided a mobile phone platform for youth to text SRH questions to a health professional, allowed adolescents to retrieve “on-demand” SRH content through a question and answer platform, and offered “push” messaging where SRH content was texted to adolescents on a regular schedule.

For example, Learning about Living’s question and answer service operates on a SMS platform, allowing young people to text their SRH and rights questions to counselors trained in gender, SRH, and human rights. Counsellors aim to reply to every question within 24 h but often respond within an average of 6 h depending on the number of questions. As Learning about Living has expanded from Nigeria to Senegal, Morocco, Egypt and Cambodia, they extended their communication platforms to also include email and Facebook. In Cambodia, Learning about Living complemented these outreach approaches by adding a mobile podcast component. Podcasts were developed by young people and consist of short audio episodes that can be delivered via recorded call to subscribers' phones, as well as through an on-demand Interactive Voice Response (IVR) call-in service. Approaches such have these have enabled Learning about Living programs to achieve tremendous reach. During the first 3 years of operation, Info Ado in Senegal answered approximately a quarter of a million SMS in total sent in by more than 67,000 individual users.

Similarly, ChatSalud in Nicaragua uses a SMS platform for health promotion aims. Chat Salud’s interactive, “ping-pong” SMS platform allows users to customize which information to read and which to skip, based on a series of menus and coded themes. Users can choose from a wide variety of SRH topics such as HIV/AIDS, sexually transmitted infections, reproductive health, and safer sexual practices.

A few projects (18% or 3 projects) used mHealth as a way to link users to needed SRH services, including family planning counseling and services, medical abortion and post-abortion care, and HIV care and treatment. For example, in 2012 MSI Ethiopia piloted an innovative electronic voucher system in order to increase demand for family planning services and counseling among young people. This mHealth approach replaced traditional paper vouchers with electronic vouchers sent directly to the client’s mobile phone. Between August 2012 and February 2013, MSI Ethiopia issued 2521 eVouchers. By the end of this period, 51% had been redeemed. Of the vouchers issued, 92% were redeemed by youth ages 15–29, which reflects not only an increase in contraceptive uptake, but also an increase in contraceptive choice, with increased reports of clients electing an IUD as their preferred contraceptive method [21].

### Scaling up ASRH mhealth interventions

These 17 projects also include several mHealth programs that began as single-country programs, but evolved their program formats and scaled to become multi-country programs with added functionalities. Learning by Living (OneWorld) began in 2007 as a sexual health internet-based platform in Nigeria. Through additional funding and in response to young peoples’ desire for mobile phone access, they added a mobile phone and additional technological components and expanded to five additional countries (Senegal, Morocco, Mali, Egypt and Cambodia) to reach more youth. Similarly, m4RH (FHI 360) began by disseminating family planning information via text messages to people of a reproductive age in Kenya and Tanzania. Based on pilot data showing high participation and resonance among young people, the project then evolved towards disseminating broader SRH information for youth ages 15–24, and has been adapted to target youth in Rwanda, Tanzania and Uganda.

## Discussion

The diversity and scale of mHealth intervention programs identified in this review reflect how healthcare professionals are increasingly leveraging mobile phones to reach adolescents with essential SRH information and services. The interventions identified for this review aimed to promote positive, preventive, and post-treatment SRH care, provide psychosocial support, and encourage use of health screening and treatment services for youth living with HIV. Mobile phones were used to increase the reach of SRH information and services to young people, particularly those living in traditionally conservative societies where sexuality and reproductive health remain highly stigmatized subjects [22, 23]. These programs leverage mobile phones to reach young people where they are and appear highly responsive to the communication channels embraced by youth in LMICs.

The small sample size of projects could be considered a limitation to this review. While there are likely additional projects that have emerged since the global call for projects was issued that would have been appropriate for inclusion in this review, the sample size of 17 projects is an accurate reflection of the projects that were offered during the data collection period.

### Challenges

mHealth is in a state of constant growth and evolution. As mHealth technologies advance and expand to improve adolescent SRH, fundamental challenges with regards to access, security, infrastructure, and functionality need to be addressed. A myriad of challenges are often presented in the early design and piloting stages [12]. For example, projects must ensure that health messaging is adapted not only to the primary languages spoken by the audience base, but also to the vernacular to which youth feel most accustomed. This process is often met with an additional design challenge: developing health messages within the character restrictions of SMS [24]. Although SMS resonates well with young people, SMS health messaging has limitations that inhibit both robust content provision and prompts for engaging users in a two-way communication. Structural issues are also present in the design and piloting stages. In resource-poor countries, a weak cellular network infrastructure or poor telecommunications service can present connectivity issues, which often compromises the continuity, reach, and quality of the intervention [25].

Maintaining the security and confidentiality of user information and data is another formidable challenge to mHealth interventions [26]. In LMICs, where mobile phones are often shared among family members, young people may have difficulty shielding information they receive or send about sensitive or taboo health topics. This challenge is related to a similar gap exemplified in the mHealth interventions listed in the table above: improving access to mHealth interventions for younger adolescents (younger than 15). Demographic data from these programs indicate that younger adolescents do not engage with mHealth messaging to the extent that older adolescents do, despite the fact that the health information provided is likely to have a more substantial impact for them. Although data is sparse, younger adolescents are less likely to own or have access to mobile phones compared to older youth, which constrains their ability to obtain health information via mobile phones.

### Future directions

Evidence from mHealth programs is becoming more robust and generating increasing data about the potential for knowledge and behavior change from mHealth interventions. While evidence suggests high feasibility, acceptability, and even knowledge and behavior change among youth participants in mobile phone interventions, these data are limited and rarely obtained using rigorous research designs and methods from youth in LMICs [27, 28]. However, two of the projects included here have been rigorously evaluated. In a randomized control trial, m4RH had a significant impact on family planning knowledge, although it was not associated with improvements in family planning use [29]. A randomized control trial for m-ASSIST found that between baseline and follow-up, anxiety decreased more (*p* = 0.013) and less emotional stress was experienced (*p* = 0.015) in the intervention group who received standard of care plus mobile messaging, compared to the group only receiving standard of care. SMSs delivered through the mobile platform were highly acceptable, with 99% of participants saying that they would recommend the messages to a friend having the same procedure [30]. Furthermore, in recent years, the field of mHealth has generated a number of research studies that highlight changes in knowledge and behavior across health topics and with a range of target audiences. Notably, this body of evidence has generated a high and rising number of studies to support systematic reviews of mobile phone interventions for health improvement [31–33].

Future mHealth interventions targeted at improving the SRH of young people should focus on assessing reductions in sexual risk behavior and uptake and maintenance of contraception and HIV services. Measuring progress on intermediate behavioral outcomes such as changes in SRH norms and increased confidence in adopting and using contraception is recommended to bolster the evidence base. In terms of optimal user engagement, future studies should also seek to address what barriers prevent younger adolescents (15 and younger) from participating more fully in mHealth interventions.

Increased evidence and programmatic guidance on the role a mHealth component should play in the larger health ecosystem, particularly in the realm of youth programming, is needed. The place that mHealth should occupy in youth programming remains undetermined. More information is needed about what proportion of resources should be allocated to a mobile phone component in combination with other youth programming elements, and whether a mHealth program can achieve success as a standalone intervention. Further, evidence is needed on how to best utilize mobile phones in youth programs to support effective interactions with health care providers and to increase service uptake among young people [12]. Finally, the cost-effectiveness of mobile phones as a communication channel in comparison to other communication formats (such as in-person counseling or community outreach events targeting young people) also has not yet been studied. In addition, more understanding is needed about the cost and cost-effectiveness of mHealth interventions for improving young people’s SRH outcomes, with respect to time and resource efficiencies when delivering SRH information and services to young people [12]. Lastly, data are needed to inform what the return is on investment with respect to knowledge, behavior, and social norm change.

## Conclusion

This review highlights many innovative, engaging, and effective uses of mobile phones to improve adolescents’ access to SRH information and services, globally. Given that the field of mhealth is still emerging, more evidence is needed on the cost, impact, and sustainability of mhealth interventions in LMIC contexts.

## Additional file

Additional file 1: The call for resources for mobile phone approaches to advance adolescent sexual and reproductive health, issued by FHI 360. (DOCX 259 kb)

## References

[1] World Bank and International Telecommunication Union (2012). The little data book on information and communication technology [Internet].

[2] Hightow-Wideman LB, Muessig KE, Bauermeister J (2015). Youth, technology, and HIV: Recent advances and future directions. HIV and Technology [Internet].

[3] Pew Research Center (2014). Emerging Nations Embrace Internet, Mobile Technology [Internet].

[4] Pew Research Center. Cell phones in Africa: Communication lifeline [Internet]. Washington, D.C: Pew Research Center; 2015 Apr 4 [cited 2015 Nov 2]. Available from: http://www.pewglobal.org/2015/04/15/cell-phones-in-africa-communication-lifeline.

[5] Kennedy EC, Harris J, Humphreys D, et al. “Be kind to young people so they feel at home”: A qualitative study of adolescents’ and service providers’ perceptions of youth-friendly sexual and reproductive health services in Vanuatu [Internet]. BMC Health Services Research. 2013;31(13). doi: 10.1186/1472-6963-13-455. cited 2015 Nov 2 10.1186/1472-6963-13-455 PMC3842673 24176059

[6] Biddlecom AE, Singh S, Woog V. Adolescents’ views of and preferences for sexual and reproductive health services in Burkina Faso, Ghana, Malawi and Uganda [Internet]. Afr J Reprod Health. 2007;11(3):99–110. Available from: https://www.ncbi.nlm.nih.gov/pmc/articles/PMC2367115/ cited 2015 Nov 2. PMC2367115 18458737

[7] Chandra-Mouli V, McCarraher DR, Phillips SJ (2014). Contraception for adolescents in low and middle income countries: needs, barriers, and access. Reprod Health.

[8] Williamson L, Wight D, Petticrew M, et al. Limits to modern contraceptive use among young women in developing countries: A systematic review of qualitative research [Internet]. Reprod Health. 2009;6(3). doi: 10.1186/1742-4755-6-3. cited 2015 Nov 2 10.1186/1742-4755-6-3 PMC2652437 19228420

[9] UNAIDS Inter-agency Task Team on Young People (2006). Preventing HIV/AIDS in young people: A systematic review of the evidence from developing countries [Internet].

[10] WHO and the Implementing Best Practices Initiative (2015). Family planning high impact practice list [Internet].

[11] Déglise C, Suggs LS, Odermatt P (2012). Short message service (SMS) applications for disease prevention in developing countries [Internet]. J Med Internet Res.

[12] Aranda-Jan CB, Dibe-Mohutsiwa N, Svelta L (2014). Systematic review on what works, what does not work and why of implementation of mobile health (mHealth) projects in Africa [Internet]. BMC Public Health.

[13] Gurman TA, Rubin SE, Roess AA (2012). Effectiveness of mHealth behavior change communication interventions in developing countries: A systematic review of the literature [Internet]. J Health Commun.

[14] Levine D, McCright J, Dobkin L (2008). SEXINFO: a sexual health text messaging service for San Francisco youth [Internet]. Am J Public Health.

[15] Gold J, Aitken CK, Dixon HG (2011). A randomised controlled trial using mobile advertising ot promote safer sex and sun safety to young people [Internet]. Health Educ Resour.

[16] Lim MS, Hocking JS, Aitken CK (2012). Impact of text and email messaging on the sexual healht of young people: A randomised controlled trial [Internet]. J Epidemiol Community Health.

[17] Vahdat HL, L’Engle KL, Plourde KF (2013). There are some questions you may not ask in a clinic: providing contraception information to young people in Kenya using SMS [Internet]. Int J Gynecol Obstet.

[18] Rodrigues R, Bogg L, Shet A (2014). Mobile phones to support adherence to antiretroviral therapy: What would it cost the Indian National AIDS Control Programme. J Int AIDS Soc.

[19] Zurovac D, Sudoi RK, Akhwale WS (2012). The effect of mobile phone text-message reminders on Kenyan health workers’ adherence to malaria treatment guidelines: a cluster randomized trial [Internet]. Lancet.

[20] Gurol-Urganci I, de Jongh T, Vodopivec-Jamsek V (2012). Mobile phone messaging for communicating results of medical investigations [Internet]. Cochrane Collaboration.

[21] Marie Stopes International (2013). eVouchers in Ethiopia Using mobile phones to increase young people’s access to contraception [Internet].

[22] One World (2013). Bila7araje Morocco [Internet].

[23] Center for Health Market Innovations (2016). MSI Papua New Guinea [Internet].

[24] Rotheram-Borus MJ, Tomlinson M, Gwegwe M, Comulada WS, Kaufman N, Keim M (2012). Diabetes buddies: peer support through a mobile phone buddy system. Diabetes Educ.

[25] Leon N, Schneider H, Daviaud E (2012). Applying a framework for assessing the health system challenges to scaling up mHealth in South Africa. BMC Med Inform Decis Mak.

[26] Haberer JE, Kiwanuka J, Nansera D, Wilson IB, Bangsberg DR (2010). Challenges in using mobile phones for collection of antiretroviral therapy adherence data in a resource-limited setting. AIDS Behav.

[27] L’Engle KL, Mangone ER, Parcesepe AM (2016). Mobile Phone Interventions for Adolescent Sexual and Reproductive Health: A Systematic Review. Pediatrics.

[28] Gonsalves L, L’Engle KL, Tamrat T, Plourde KF, Mangone ER, Agarwal S (2015). Adolescent/Youth Reproductive Mobile Access and Delivery Initiative for Love and Life Outcomes (ARMADILLO) Study: formative protocol for mHealth platform development and piloting. Reprod Health.

[29] Johnson D (2016). A randomized controlled trial of the impact of a family planning mHealth service on knowledge and use of contraception. Contraception.

[30] Constant D, de Tolly K, Harries J, Myer L (2014). Mobile phone messages to provide support to women during the home phase of medical abortion in South Africa: a randomised controlled trial. Contraception.

[31] Hall AK, Cole-Lewis H, Bernhardt JM (2015). Mobile text messaging for health: a systematic review of reviews. Annu Rev Public Health.

[32] Higgs ES, Goldberg AB, Labrique AB, Cook SH, Schmid C, Cole CF (2014). Understanding the role of mHealth and other media interventions for behavior change to enhance child survival and development in low- and middle-income countries: an evidence review. J Health Commun.

[33] Orr JA, King RJ (2015). Mobile phone SMS messages can enhance healthy behaviour: a meta-analysis of randomised controlled trials. Health Psychol Rev.

