# Supplementary material for: Meet us on the phone: mobile phone programs for adolescent sexual and reproductive health in low-to-middle income countries
Source: Reprod Health. 2017 Jan 17;14:11. doi: 10.1186/s12978-016-0276-z (PMC5240300; doi:10.1186/s12978-016-0276-z)

Call for resources on mobile phone approaches for adolescent sexual and reproductive health


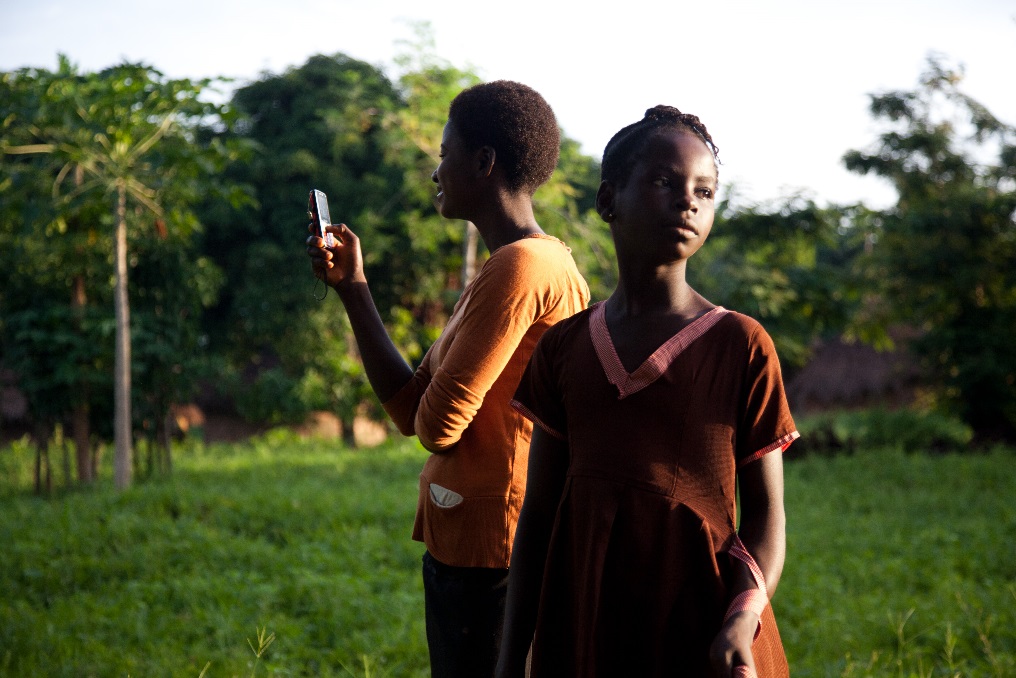
The WHO mHealth Technical and Evidence Review Group (mTERG) in collaboration with FHI 360 is issuing a call for project descriptions and associated resources on mobile phone approaches for addressing adolescent sexual and reproductive health (ASRH).

©STARS/Kristian Buus

We are requesting these materials to inform a review of innovative approaches in this field. Relevant materials can include:

- Descriptions of projects
- Slide decks describing project or project results
- Case studies or briefs
- Other project overview documents
- Project reports
- Published peer reviewed manuscripts

The global health community is embracing innovative approaches to ASRH including mhealth. We are eager to learn more about the creative and cutting edge approaches that partners and countries are utilizing to improve ASRH. We greatly appreciate your taking the time to share your innovative projects and resources!

Please send your materials to **kplourde@fhi360.org** by July 30, 2014.


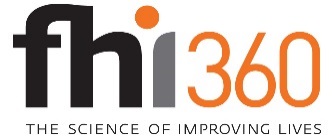

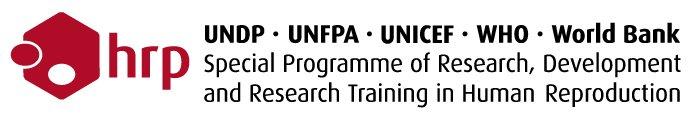

Supplement: Additional file 1: — The call for resources for mobile phone approaches to advance adolescent sexual and reproductive health, issued by FHI 360. (DOCX 259 kb) [file 12978_2016_276_MOESM1_ESM.docx]
